# Supplementary material for: Reducing annotation burden in physical activity research using vision language models
Source: Sci Rep. 2025 Oct 24;15:37253. doi: 10.1038/s41598-025-21350-6 (PMC12552446; doi:10.1038/s41598-025-21350-6)
Supplement: Supplementary file 1 — Supplementary Information. [file 41598_2025_21350_MOESM1_ESM.pdf]

Reducing Annotation Burden in Physical Activity Research Using Vision Language Models

Abram Schönfeldt
Department of Population Health, University of Oxford, Oxford, United Kingdom

Benjamin Maylor
Department of Population Health, University of Oxford, Oxford, United Kingdom

Xiaofang Chen
School of Epidemiology and Health Statistics, Chengdu Medical College, Sichuan, China

Ronald Clark
Department of Computer Science, University of Oxford, Oxford, United Kingdom

Aiden Doherty
Department of Population Health, University of Oxford, Oxford, United Kingdom

Section 1 includes additional details when mapping the labels to activity intensity classes. Section 2 goes into more detail on the properties of the validation studies used in this work, and Section 3 provides additional implementation details. Section 4 shows confusion matrices of the best models, and illustrates examples of generated captions mapped to different activity classes, and Section 4.4 presents median κ scores of one annotator confined to predicting activity intensity from single images on a subset of the data.

1 Mapping compendium annotations to activity intensity classes

This mapping from the applied compendium of physical activity labels to activity intensity classes was originally done in [1]. Note, however, that the published dictionary does not strictly abide by these definitions, since some activities which technically would be MVPA, such as "Cleaning, sweeping carpet or floors, general", MET = 3.3, were mapped to LIPA based on the discretion of the authors. To be consistent with previous work using the Oxfordshire study, we used this mapping, also applying it to the labels in the Sichuan study accounted for by it.

There were some labels used in the Sichuan study not included in the dictionary from [2]. To address these, an updated dictionary was created using the 2024 compendium of physical activity [3] by matching the raw labels to their updated entries using their activity codes. This dictionary provides an updated mapping from the raw labels from both validation studies to activity intensity classes, and the latest entries in the compendium of physical activity and will be made available with the supplementary material.

2 Properties of two free-living, egocentric timelapse

The two studies used in this work used wearable cameras capturing sparse sequences of images to label activities of daily living. Figure S1 provides an example of a sequence of activities captured by a wearable camera with a time interval of 20 seconds between consecutive frames. At this frame

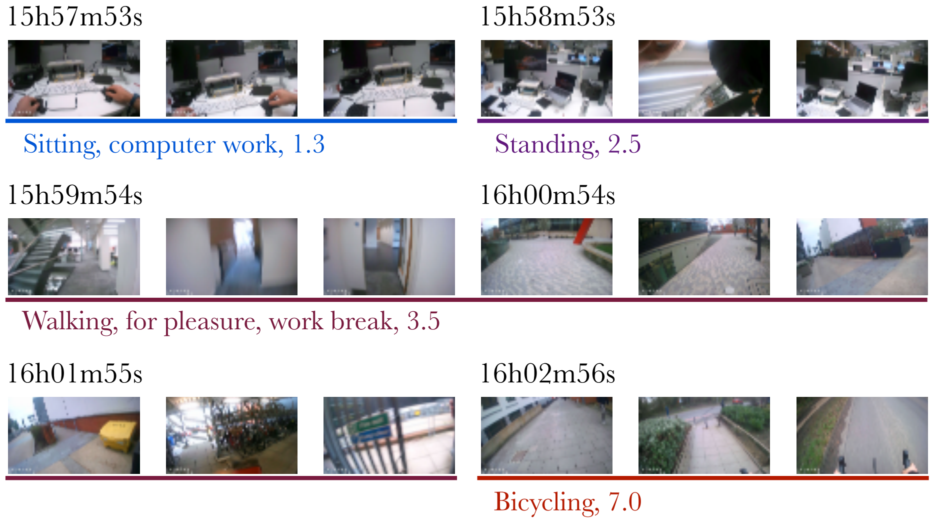

Figure S1: A sequence of images captured with an interval of 20 seconds between frames, labelled with activities and MET values.

Table S1: Percentage of images labelled as uncodeable, unknown or undefined.

	Oxfordshire	Sichuan
`uncodeable;0002 image dark/blurred/obscured`	16.40%	56.98%
`uncodeable;0001 camera taken off`	1.68%	6.91%
`undefined`	0.17%	0.04%
`<unknown>`	0.01%	0.00%

rate, the transition between environments can be abrupt, and the segment of cycling only becomes apparent once the handlebars are visible a few frames after the start of the event.

Figure S2a shows the relationship between the median time between images and the number of labelled events per participant. The median time differences for the participants in the Oxfordshire study are clustered in 4 bands with the two most prominent clusters located around 20s, compared to the Sichuan study, whose participants are clustered in a band located at a median time of around 80s. There does not seem to be a strong relationship between these variables, since at fixed median time between images, we observe a large variation in the number of labelled events, though intuitively, at extremely low time intervals it is likely that many brief activities are missed, and it becomes impossible to accurately distinguish the timing of events. Figure S2b shows quartile plots of the frequency of each label per participant. In addition to the class imbalance, this shows the large range in the prevalence of the classes across participants.

Finally, Figure S2c, which is a scatter plot of images with the x-coordinate showing the mean pixel value of each image as a proxy for how dark it is, and the y-coordinate the variance in the pixel values as a proxy for how dynamic it is, illustrates the many obscure unlabelled images. Only 74% of the images in the Oxfordshire study, and a much lower 34% of the images in the Sichuan study were labelled with non-trivial labels. There were a few ways annotators expressed that they were unable to label images, including “image dark/blurred/obscured”, “camera taken off”, “undefined”and “unknown”. Table S1 shows the percentage of the images which could not be labelled for a particular reason. For completeness, which were simply not labelled. In the main text, we take labelled to mean an image has a non-trivial label.

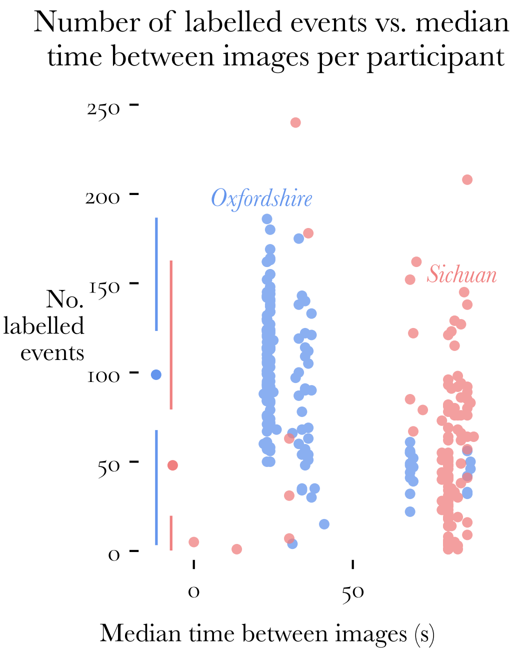

(a) Scatter plot showing that most participants within the Oxfordshire study had a lower median time between images compared to participants within the Sichuan study, as well as more labelled events.

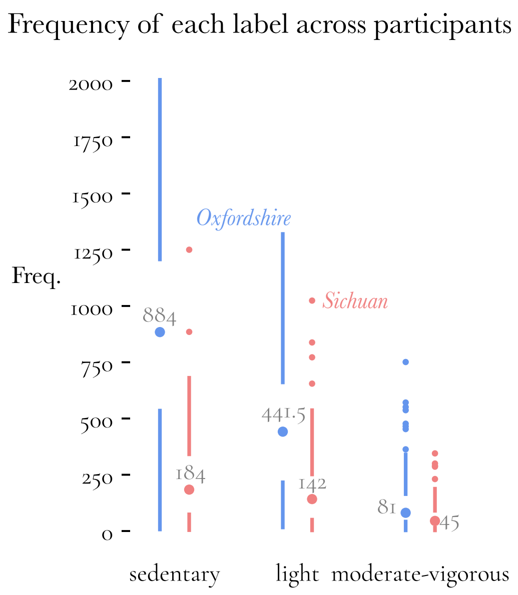

(b) Quartile plot showing the imbalance in the prevalence of the activity intensity labels, and the relatively low number of instances of each label in the Sichuan study versus the Oxfordshire study.

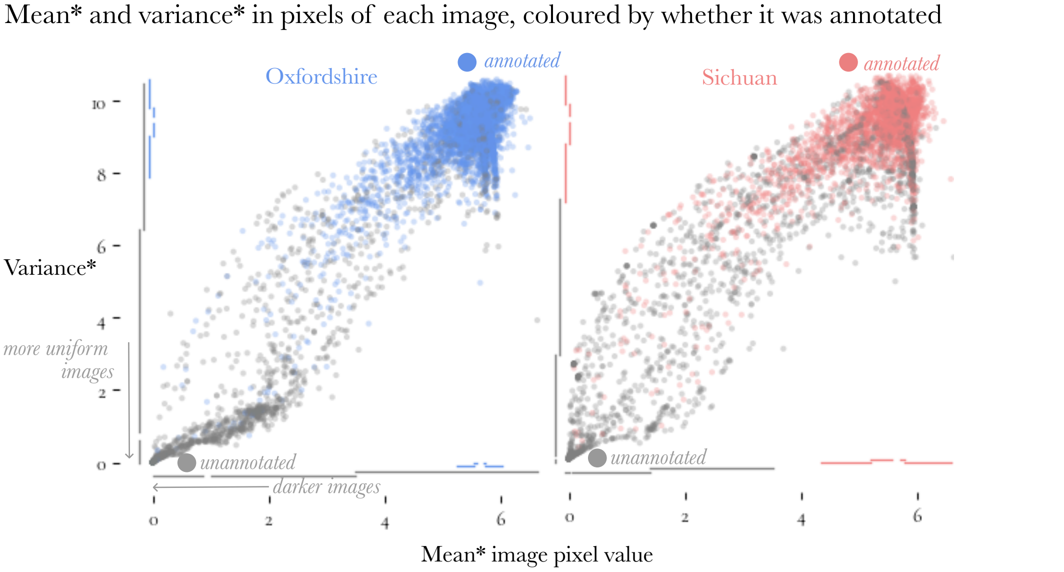

(c) Scatter plot illustrating the relationship between unannotated images and images with low mean* and variance* pixel values. The mean* of the pixel values in each image was calculated as $\log(1 + \sum_c \mu_c)$, where μ_c represents the mean of pixel values in an RGB image in channel c, and the variance* in the pixel values is an analogous transformation of the per channel variances. Intuitively, darker images will have lower mean pixel values, and images which are uniformly grey (or any other colour), will have no variance in their pixel values.

Figure S2: Visualisation of the temporal sparsity of images, the label imbalance, and the large number of obscure images in the Oxfordshire and Sichuan validation study. The median participants day has 100 labelled events in the Oxfordshire study, versus 50 in the Sichuan study, with the much lower capture rate in this study potentially limiting the number of events that could be labelled. The majority of images were labelled as depicting sedentary activity.

Table S2: Huggingface model IDs, number of parameters and size of each model.

Zero-shot models	Huggingface model ID	No. parameters (millions)
CLIP	openai/clip-vit-large-patch14	428
BLIP2	Salesforce/blip2-flan-t5-xl	3 942
LLaVA	llava-hf/llava-1.5-7b-hf	7 063
Fine-tuned models		
ResNet-50	IMAGENET1K_V2	25
ViT (CLIP image encoder)	openai/clip-vit-large-patch14	304

Note: For the ResNet, we used the torchvision ImageNet1K V2 checkpoint [4] .

Table S3: Hyperparameters tuned for each model.

Hyperparameter	Values
`mapping approach`	direct, via clean
`new tokens`	5,10,20,40
`prompt`	...
`reword labels`	true, false
`batch size`	32, 64, 128, 256, 512
`finetune`	last layer, full model
`learning rate`	$10^{-i}, i \sim U(1, 5)$
`trivial augment`	true, false
Zero-shot models	Hyperparameters tuned
CLIP	`mapping approach`
BLIP2	`mapping approach`, `new tokens`, `prompt`, `reword labels`
LLaVA	`mapping approach`, `new tokens`, `prompt`, `reword labels`
Fine-tuned models	
ResNet	`finetune`, `learning rate`, `batch size`, `trivial augment`
ViT	`finetune`, `learning rate`, `batch size`, `trivial augment`
ResNet-LSTM	`learning rate`*

Note: We only tried three different learning rates for the ResNet-LSTM, $l \in \{10^{-3}, 10^{-4}, 10^{-5}\}$.

3 Implementation

Table S2 gives the Hugging Face model IDs for the models used in this work, as well as the model sizes. Models weights were represented using 16-bit floating point precision (torch.float16), and were able to run on a single Tesla V100 with 32GiB of VRAM. Table S3 shows the hyperparameters tuned for each model. For the generative models, `reword labels` controlled whether the text representations for sedentary behaviour, LIPA and MVPA were "sedentary", "light", "MVPA", or ""sedentary behavior", "light physical activity", and "moderate-to-vigorous physical activity". The set of prompts were too long to include in the table and are listed in the configuration files in the repository.

4 Additional results

4.1 Confusion matrices

Figure S3 shows confusion matrices for the best checkpoint for LLaVA and ViT. These confusion matrices ignore variation in performance at the participant level, though facilitate comparisons to work by [5]. We include the converted confusion matrix from this work in Table S4.

4.2 Semantic mistakes

Sometimes, the captions produced by the generative models were mapped to labels that did not mean the same thing as the produced caption. In Table S5, we give examples of the produced captions, the label they were ultimately mapped to, possibly via an intermediate clean label, as well as the

Table S4: Confusion matrix from Table 3 of [5], showing the performance of XGBoost [6] based on features from AlphaPose [7] with the rows and columns related to moderate and vigorous physical activity combined.

True / Predicted	Sedentary	Light	MVPA
Sedentary	13 259	4 915	345
LIPA	197	939	129
MVPA	1255	2427	6594

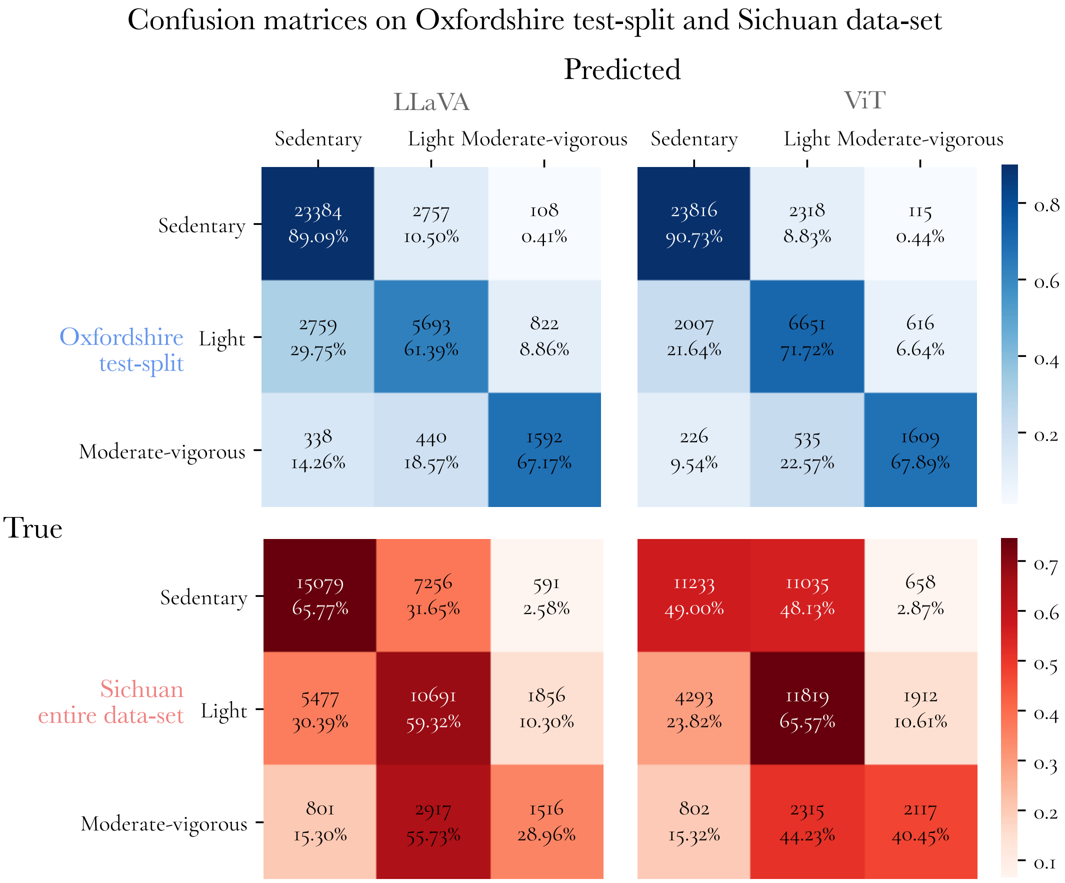

Figure S3: Confusion matrices showing the disagreements between the human and model predictions, for LLaVA and ViT, particularly on the Sichuan study. The percentages (and colours) are normalised based on the total number of "true" instances of each label.

similarity score from the sentence embedding model. In our main results, we additionally observed that the best performing VLM was prompted "Walking, Running, Sitting, Standing, Other. Based on the objects in the image, what is the person likely doing?", which then had its response directly mapped to one of the activity intensity classes.

These results suggest that the VLMs are not performing this task in the same way as trained human annotators, who would not make these obvious semantic errors, e.g. equating "a woman playing with a frisbee" with "bowling" (a light intensity physical activity). In theory, a human can walk with range of different intensities, and relying on a text description of walking alone to infer activity intensity reveals further limitations with this approach.

4.3 Poor performance in poor light

Wearable cameras used in free-living settings frequently produce dark images where the camera view is fully or partially obscured, such as by clothing. Figure S2c, shows that dark, uniform images tended

Table S5: Examples of the raw captions produced by different prompts and models, and the labels they were mapped to. Some of these captions were first mapped via one of the clean labels associated with each coarse label.

Caption →	Mapped via →	Mapped to	Sim.	
a woman sitting in a chair and talking to a woman	sitting meeting or talking with others	sedentary behaviour	0.47	✓
a fence and a yard	mowing lawn	MVPA	0.41	?
a woman playing with a frisbee	bowling	LIPA	0.32	✗

Table S6: Model performance on images grouped by brightness percentiles. Images in the 0-5 percentile represent images in the darkest 5% of the Oxfordshire test-set.

Percentile Range	Model	Median κ	Quartile 1-3
Extreme ends (0–5, 5–95, 95–100 percentiles)			
0–5	ViT	0.33	0.18–0.55
5–95	ViT	0.68	0.62–0.74
95–100	ViT	0.56	0.35–0.83
0–5	LLaVA	0.31	0.12–0.44
5–95	LLaVA	0.53	0.48–0.65
95–100	LLaVA	0.44	0.27–0.66
Broader groups (0–25, 25–75, 75–100 percentiles)			
0–25	ViT	0.64	0.51–0.66
25–75	ViT	0.69	0.62–0.77
75–100	ViT	0.65	0.58–0.73
0–25	LLaVA	0.51	0.43–0.56
25–75	LLaVA	0.58	0.43–0.65
75–100	LLaVA	0.57	0.42–0.61

to be left unannotated by human annotators. To investigate whether lighting similarly impacted model performance, we looked at model performance on images grouped by brightness level (Table S6). We calculated the mean pixel value in each image, and aggregated per-participant performance in the ends of the image brightness sample distribution in the test-set. Both models performed substantially worse in the darkest 5% of the images. However, extending the analysis to the darkest 25% of the images revealed performance only slightly below that of the middle range, confirming that this decline in performance is in this extreme ends of the distribution.

4.4 Human performance from single images

To estimate human performance for labelling activity intensity from single images, one of the authors (Abram Schönfeldt) manually labelled $\geq$ 500 images from participants in the test splits from the Oxfordshire (25 participants) and Sichuan (13 participants) validation studies. The images were sampled uniformly at random and presented without temporal context, which is not how these datasets were originally labelled, though reflects the information seen by the models. The median κ (1st, 3rd quartile) on the Oxfordshire test-split was 0.636 (0.457, 0.722), and 0.572 (0.464, 0.610) on the Sichuan study. Though limited by the small amount of labelled data, and single annotator, these results suggest that the current model performance might be similar to human performance.

References

[1] Rosemary Walmsley, Shing Chan, Karl Smith-Byrne, Rema Ramakrishnan, Mark Woodward, Kazem Rahimi, Terence Dwyer, Derrick Bennett, and Aiden Doherty. Reallocation of time between device-measured movement behaviours and risk of incident cardiovascular disease. *British journal of sports medicine*, 56(18):1008–1017, 2022.

[2] Shing Chan, Yuan Hang, Catherine Tong, Aidan Acquah, Abram Schonfeldt, Jonathan Gershuny, and Aiden Doherty. Capture-24: A large dataset of wrist-worn activity tracker data collected in the wild for human activity recognition. *Scientific Data*, 11(1):1135, 2024.

[3] Stephen D Herrmann, Erik A Willis, Barbara E Ainsworth, Tiago V Barreira, Mary Hastert, Chelsea L Kracht, John M Schuna Jr, Zhenghua Cai, Minghui Quan, Catrine Tudor-Locke, et al. 2024 adult compendium of physical activities: A third update of the energy costs of human activities. *Journal of Sport and Health Science*, 13(1):6–12, 2024.

[4] Adam Paszke, Sam Gross, Francisco Massa, Adam Lerer, James Bradbury, Gregory Chanan, Trevor Killeen, Zeming Lin, Natalia Gimelshein, Luca Antiga, et al. Pytorch: An imperative style, high-performance deep learning library. *Advances in neural information processing systems*, 32, 2019.

[5] Sarah Kozey Keadle, Skylar Eglowski, Katie Ylarregui, Scott J Strath, Julian Martinez, Alex Dekhtyar, and Vadim Kagan. Using computer vision to annotate video-recoded direct observation of physical behavior. *Sensors*, 24(7):2359, 2024.

[6] Tianqi Chen and Carlos Guestrin. Xgboost: A scalable tree boosting system. In *Proceedings of the 22nd acm sigkdd international conference on knowledge discovery and data mining*, pages 785–794, 2016.

[7] Hao-Shu Fang, Jiefeng Li, Hongyang Tang, Chao Xu, Haoyi Zhu, Yuliang Xiu, Yong-Lu Li, and Cewu Lu. Alphapose: Whole-body regional multi-person pose estimation and tracking in real-time. *IEEE Transactions on Pattern Analysis and Machine Intelligence*, 45(6):7157–7173, 2022.
